# Testing an Animal Welfare Assessment Protocol for Growing-Rabbits Reared for Meat Production Based on the Welfare Quality Approach

**DOI:** 10.3390/ani10081415

**Published:** 2020-08-13

**Authors:** Nadina Botelho, Madalena Vieira-Pinto, Pau Batchelli, Joaquim Pallisera, Antoni Dalmau

**Affiliations:** 1 Department of Veterinary Sciences, Universidade de Trás-os-Montes e Alto Douro (CECAV, UTAD), 5000-801 Vila Real, Portugal; nbotelopes@gmail.com (N.B.); mmvpinto@utad.pt (M.V.-P.); 2 Department of Animal Welfare, Institute of Agri-food Research and Technology (IRTA), Veinat de Sies s/n., 17121 Monells, Spain; pau.batchelli@irta.es (P.B.); Joaquim.pallisera@irta.es (J.P.)

**Keywords:** animal welfare, appropriate behaviour, assessment protocol, good feeding, good housing, good health, growing-rabbits

## Abstract

**Simple Summary:**

Animal welfare assessment protocols allow one to understand the level of welfare and to identify the main parameters to improve on farms. Accordingly, farmers have the possibility to use these tools for implementing corrective measures to improve animal welfare. This study was performed through the application of an animal welfare assessment protocol for growing-rabbits in 32 farms from Spain and Portugal. The scores obtained ranged from 44 to 82 points out of 100, depending on the farm, showing a good discriminative capacity among the 32 farms assessed. However, some key points for improving were identified in most of the farms. These points were to provide more space for the animals, improve the protocols for emergency killing due to health problems or any other cause, and to provide the farmers proper training in animal welfare.

**Abstract:**

The objective of the present study is to present an animal welfare assessment protocol for growing-rabbits for discussion after its implementation in 32 farms from Spain and Portugal. The protocol comprises the principles of Good Feeding, Good Housing, Good Health and Appropriate Behaviour of the Welfare Quality protocols and includes 36 welfare parameters. Overall, the protocol showed a good capacity for discrimination between farms, with scores ranging 44 to 82 points. The protocol seems reliable for the assessment of animal welfare after proper training of auditors. However, for the criteria social behaviour and other behaviours, further research is needed to ascertain if the methodology and times of observation used are appropriate. Some farms had high mortality rates with a low prevalence of health problems, while others had low mortality rates with high prevalence of health problems due to different managements of culling. The protocol should be improved, to impede farms with high mortality rates but a low prevalence of health issues the day of the audit from obtaining better scores than the second type of farms, by limiting the compensation in key measures. The main points to be solved in the growing-rabbit farms were: to provide more space to the animals; register the number of animals culled accurately; change cervical dislocation for another killing method and provide the farmers training in animal welfare.

## 1. Introduction

In order to understand the concept of animal welfare, the OIE (World Organisation for Animal Health) states that a good level of welfare exists when the animal is healthy, comfortable, well-nourished, safe and capable of expressing innate behaviour without suffering, pain, fear or anguish. In the last few decades, animal welfare has been a growing concern for society. In this regard, the European Commission carried out a study by surveying 27,672 European consumers, of which 94% stated that it is important to defend animal welfare on farms, 64% stated that they would like to obtain more information about animal production conditions, and 59% mentioned that they are willing to pay more for products that respect animal welfare [1].

The consumption of meat from rabbits has reduced in Spain and Portugal in the last few years (i.e., the Portuguese Association of Rabbits Cuniculture estimates a reduction of 16% in the consumption of this meat in Portugal in the last 10 years), and one of the causes could be the way that citizens see this production system. On the other hand, according to the FAOSTAT (Food and Agriculture Organization of the United Nations data), from 2008 to 2018, the number of rabbits reared in the world for production increased by 9.78% [2]. In consequence, due to the increasing demands for better production systems of the internal markets and the increased competition when accessing external markets, producers are more and more motivated in considering animal welfare as a key factor in their production systems.

Although in terms of kg of animals produced in Europe or in the world, rabbits are negligible in comparison to other species; animal welfare considers individuals and not tonnes of meat. According to the FAOSTAT, in 2018, the number of slaughtered animals in the world for meat production was of 68.785 billion chickens, 1.484 billion pigs, 922 million rabbits, 656 million turkeys, 573 million sheep, 479 million goats, 302 million cattle and 5 million horses [2]. Therefore, rabbits are the third species in number of heads reared for meat production in the world. Nevertheless, the interest for the welfare of rabbits as animals reared for meat production has elicited less interest than for other species. For instance, from 2004 to 2009 the EU funded one of the most ambitious projects on animal welfare ever developed, the Welfare Quality project. One of the aims of this project was to develop protocols to assess animal welfare in an objective, science-based and practically applicable way, focusing the assessment on animal-based parameters [3]. However, this project was focused only on cattle, pigs and chickens. After this project, and following the principles stated by the Welfare Quality, the EU funded a second project, named AWIN (Animal Welfare Indicators), that covered the forgotten species in the previous one: turkeys, sheep, goats and horses. Rabbits were never considered in either of the two European projects. However, funded by the Ministry of Economic Affairs, Agriculture and Innovation of the Netherlands, a first step in the development of an assessment protocol for commercially-housed rabbits was made [4]. This consisted of describing possible parameters for the different criteria and principles as used in Welfare Quality. This was done by combining the scant information present in the literature and the opinion of experts from different countries. This basis was tested in Spain to build a possible protocol for growing-rabbits and, from 2016 to 2018, it was tested in different rabbit farms for meat and fur production. According to the results obtained, some additional parameters were added, and others changed when needed. Then, the thresholds for the different parameters and a score system were developed.

The objective of the present study is to present the protocol based on the Welfare Quality approach and the basis developed by De Jong et al. (2011) [4] for discussion, after its implementation in the 32 farms that were assessed in Spain and Portugal.

## 2. Materials and Methods

The study was approved by the Institutional Animal and Use Committee (IACUC) of IRTA (Institute of Agrifood Research and Technology) with the note: “Experimental research performed in commercial farms with no intervention on the animals”.

Twenty Spanish and 12 Portuguese growing-rabbit farms, with conventional cages organized in rows inside pavilions, were visited between October 2018 and November 2019 and assessed by means of an animal welfare protocol during a single visit. The assessment was carried out by two different auditors, one in Spain and one in Portugal, who were trained with the same methodology and tested against a gold standard following the training procedure established in the Welfare Quality [3]. The Spanish farms were located across the entire country and in all cases they were in the first year of an animal welfare certification program, while the Portuguese farms were in the region of Trás-os-Montes e Alto Douro, Porto and Beira Alta and were not included in any animal welfare certification program. The assessment was made in most of the cases when the animals were around 50 to 60 days old, a few days before being slaughtered, as the objective of the assessment protocol is to assess the accumulative state of the animal at the end of their stay in the housing facilities. Cages to be assessed were selected randomly throughout the buildings to be representative of the overall picture of the farm. Farms contained between 765 to 17,021 growing-rabbits.

The Welfare Quality scheme provides four different principles to assess animal welfare, and these are divided into 12 criteria (Table 1). This is the structure used in the present study. However, as in the present protocol it was not possible to identify any good parameters to assess the positive emotional-state criterion, the protocol therefore includes only 11 criteria. Good Feeding includes six parameters (one animal-based); Good housing includes eight parameters (two animal-based), Good Health includes 18 parameters (13 animal-based) and Appropriate Behaviour contains four parameters (three animal-based). The set of parameters selected were based on research by De Jong et al. (2011) [4]. However, after a first discussion with a group of five experts in rabbit production and animal welfare, some of the parameters were not considered and others were included. For the good feeding principle, the percentage of emaciated rabbits at the slaughter plant was discarded because of feasibility reasons, as it would have been necessary to visit the slaughterhouse. In contrast, the parameter, feeder cleanliness, was added to the list proposed by De Jong et al. (2011) [4]. For good housing, the parameter, fully stretched lying in the pen or at the elevated platform or shelter, was considered suitable for does, but not for growing-rabbits and simultaneous resting was also considered not suitable for growing-rabbits because of the interference with fearfulness when the auditor approached the cage. The parameters, presence of shelter and elevated platform, and size of elevated platform and shelter, were considered more important in does than in growing-rabbits by the experts, as they can reduce the available height for the animals during the growing phase and increase dirtiness of animals below the platform. Floor type and quality of littered floor was considered as well as being important for does and kits, but not for growing-rabbits. In the study by De Jong et al. (2011) [4], the stocking density and cage size were included in comfort around resting and ease of movement. However, according to the Welfare Quality [3], one measure should stay in only one criteria and never in more than one, so it was decided to include the stocking density and height of the cage only in the ease of movement criterion. Lame animal was included in ease of movement by De Jong et al. (2011) [4], but in the present protocol it is considered in absence of injuries. The parameters respiration rate, red ears and free movement (animals hopping, jumping, turning and running) were included initially but discarded later after being tested on farm due to lack of feasibility. Finally, three parameters that will be defined later: wet and dirty animals and burning hair, not considered by De Jong et al. (2011) [4], were included.

For good health, the parameter technical performance was discarded by experts due to the difficulties in having a standard, and most of the management procedures proposed were considered only suitable for does, but cleanliness of the facilities was included in the protocol. The parameters toes damaged, and diarrhea and teeth abnormalities were included initially, but discarded later during first tests on farms, as of all them required the animals to be caught and this was stressful for the animals and reduced the feasibility of the protocol. In contrast, the parameters fallen ears, gait score, risk of injuries, fly presence and emergency killing, not considered by De Jong et al. (2011) [4], were included. For appropriate behaviour, parameters already included in other principles, such as wounds on the body, hopping, coat condition or height of the cage were not considered. In addition, for the human approach and novel object tests, a modification of the test proposed by Hansen and Moller (2001) [5] for minks maintained in cages was used. However, its use was discarded in growing-rabbits due to the difficulties in doing the test in cages with more than one individual. The use of enrichment material and an elevated platform were considered for adult animals, but not for growing-rabbits, so these too were discarded. The parameters kit mortality, nesting material and nest quality, were appropriate for breeders, but not for growing-rabbits. Finally, it was considered that further research is needed for the parameter called description of the behaviour of a group before its inclusion in a protocol. On the other hand, the parameters isolated animals and training of personnel on animal welfare, were included in this principle.

Once the protocol had been defined, seven different farms were visited to obtain data (three of these were visited twice), and five of the ten assessments were performed at the same time by two auditors. These visits were used to refine the individual parameters to increase repeatability between auditors, to detect problems of feasibility (as mentioned above some measures were discarded after these tests) and to decide the best strategy for sampling. Then, the data were presented to the same first five experts for discussion. After checking the real results found in the farms, the experts individually proposed the thresholds for each measure, the weight of each measure inside each criterion, the weight of each criterion inside each principle and the weight of each principle in the overall score. These individual exercises were then discussed as a group until reaching an agreement. Unless otherwise stated, animal-based measures were assessed in 2% to 4% of animals on each farm, whereas resource-based measures were assessed in 3% to 5% of cages on each farm. In fact, auditor one used a mean of 300 animals per farm per clinical measure and 50 cages for resources, while auditor two used a mean of 170 animals per farm per clinical measure and 20 cages for resources. These numbers are based on the calculations performed for the Welfare Quality protocols, where 100 individuals were assessed for laying hens and 150 for broilers and pigs for clinical measures [6,7].

### 2.1. Good Feeding

The Good Feeding principle is assessed by means of the combination of two criteria: absence of prolonged hunger (65% of the total score) and absence of prolonged thirst (35% of the total score; Table 2). Inside this principle, only one animal-based measure is used: body condition. The way to assess body condition is based on research by Popescu et al. (2013) [8], where five categories were considered (emaciated, lean, ideal, fat and obese), and simplified according to the Welfare Quality (2009) [6,7], with just two categories, good (ideal) and lean (lean and emaciated). The body condition parameter was assessed along with the parameters of animals per feeder, cleanliness of feeders, animals per drinker, functioning of the drinkers and cleanliness of the drinkers.

### 2.2. Good Housing

The Good Housing principle is assessed by means of the combination of three criteria: comfort around resting (35%), thermal comfort (15%) and ease of movement (50% of the total score; Table 3). A wet animal is considered when any part of the fur is wet. For dirtiness, two categories are considered. The animal is scored as moderately dirty when from 10% to 30% of the body is dirty, and severely dirty when more than 30% of the body is dirty. The parameters of dust, light quality, environmental temperatures and burning hair were assessed across all of the facilities in each farm. The dust parameter is assessed by means of a black surface of approximately 10 × 15 cm and left during the assessment at the center of the building housing the rabbits at the same height as their heads. At the end of the assessment, the level of dust accumulated is assessed considering three possibilities: no evidence of dust; minimal evidence of dust (a thin covering of dust) and a lot of dust (possible to write on the paper with a finger) or paper not visible. The quality of light is considered correct when it is possible to check all of the animals with no problems and if at least 8 h of light and 8 h of darkness are provided to the animals. This parameter does not add points when it is found correct, but it can subtract 20 points from the comfort around resting criterion when it is found to not be acceptable. The temperature parameter is assessed according to the registers of the temperature in the farm. In case there are no registers, zero points are given. If there are registers, excellent is given when the temperatures, maximum and minimum, range from 1 °C to 28 °C. Acceptable is considered when, up to two days in the last three months, the temperature registered is outside of this range. The burning hair parameter is related to the burning of hair accumulated in the cages for improving environmental conditions. Nevertheless, if the temperature is outside of the proposed range (1 °C to 28 °C) during this practice, or the temperature is not registered during this practice, 20 points will be subtracted from the thermal comfort criterion. To evaluate the “ease of movement” criterion, the height of the cage and stocking density parameters were assessed.

### 2.3. Good Health

The Good Health principle is assessed by means of the combination of three criteria: absence of injuries (40%), absence of diseases (40%) and absence of pain induced by management (20%; Table 4). For wounds on the body, a lesion is considered a fresh scratch or open lesion larger than 2 cm in any part of the animal and that has not healed. Any animal with these lesions is assessed as moderately injured. However, in the case of a lesion of more than 5 cm, the animal is assessed as severely injured. For wounds on the ears, no distinction for size is made, and only lesions of more than 2 cm are considered. Nonetheless, in this case, old lesions are distinguished from fresh lesions. Fallen ears are considered as just the absence or presence of the problem, and the worse one of the two ears is considered. For hairless areas, only those with a size of at least 2 cm are considered. Gait score considers three possibilities: no problems, if the animal does not have any difficulty in moving; moderate problem, if the animal has any difficulty in moving and severe problem, if the animal has several difficulties (no use of one leg or minimum weight-bearing). Nasal and ocular discharge considers only the presence or absence of discharges in the animals, although signs of conjunctivitis are also considered as present for ocular discharge. Coughing and sneezing was assessed by observing a minimum of 10 cages for two minutes and considering three possibilities: no problems, if no coughing or sneezing is registered; moderate problems, when no more than two events (coughing or sneezing) is registered in a cage; and severe problems, when 3 or more events are detected in a cage. Any sign of inflammation of the skin is considered, as present or absent, inside the parameter labelled dermatophytosis, dermatitis or abscesses. For neck torsion, three conditions are considered: absence, when the neck is perfect; moderate problem, when the animal has a neck torsion but is able to eat and drink with no difficulties, and severe problem, when the neck torsion makes the access to food and water very difficult for the animal. Mange is assessed as presence or absence. In the case of absence, it is not considered for the overall score, but in the case of being present in only one animal, 20 points will be subtracted from the absence of disease criterion. Mortality and culling rates are assessed according to the records of the farm for at least the previous three months. The farmer is asked about the procedure for emergency killing and, when possible, this is assessed during the visit according to the possibilities established in Table 4. Risk of injuries assesses the risk for the animals to be injured by bad maintenance of the cages or other elements in their surroundings. This parameter is not considered for the score if the result is absence of problems, but it subtracts 15 points from the absence of injuries criterion if one cage with a problem is found and 30 points if more than one cage with a problem is found. The cleanliness of the housing system parameter has three possibilities: the cage is clean; the cage is partly dirty, when only a part of the cage is not clean (including a lot of presence of hair, compacted dry food and mold) and dirty cage, when all of the cage is very dirty. If there are no problems, this parameter is not considered for the assessment, but depending on the number of partly dirty or dirty cages, this can take 10 or 20 points from the absence of diseases criterion. Something similar happens with the presence of flies, where three possibilities are possible: no presence of flies or fly eggs, when the parameter is not used for the final score; presence of flies or fly eggs, when 10 points are subtracted from the absence of disease criterion, and presence of flies and fly eggs, when 20 points are subtracted from the criterion.

### 2.4. Appropriate Behaviour

The Appropriate Behaviour principle is assessed by means of the combination of three criteria: social behaviour (35%), other behaviours (35%) and human–animal relationship (30%; Table 5). The parameters of negative social behavior, isolated animals and abnormal behaviour were assessed in 10 cages for a period of two minutes each at the same time as coughing and sneezing. For negative social behaviour, as such any event in which an animal is biting another one is considered. Isolation is considered when one animal is housed alone and has no visual contact with any other animal. Abnormal behaviour consists of animals scratching or biting the cage or performing repetitive behaviours without an apparent objective (stereotypies). The training parameter was assessed by asking the farmer for certificates of attendance for him and his personnel of a training course on animal welfare, and it considers three levels: all personnel in the farm in contact with the animals are trained on animal welfare; at least one of the persons in contact with the animals is trained in animal welfare, and none of the persons in contact with the animals in the farm is trained in animal welfare.

### 2.5. Overall Assessment

The Welfare Quality provides a final score for a farm as a result of the combination of the scores of the different principles. When the final score is between 0 and 19, the farm is considered not classified. When the final score is between 20 and 54 points the farm is considered acceptable. When the final score is between 55 and 79 points the farm is considered enhanced and, finally, a farm from 80 to 100 points is considered excellent (Welfare Quality, 2009) [6]. The score “no classified” is considered as not acceptable in the present protocol. For an overall assessment of a farm, the four principles of the growing-rabbit protocol have different weights because of the number or importance of the parameters included in the specific principles (6, 8, 18 and 4 parameters, respectively). To obtain the final score of a farm, 15% of the score depends on the Good Feeding principle, 30% from the Good Housing principle, 35% from the Good Health principle and 20% from the Appropriate Behaviour principle.

## 3. Results

### 3.1. Good Feeding

The Good Feeding principle is assessed by means of the combination of two criteria: absence of prolonged hunger and absence of prolonged thirst.

Inside the criterion of absence of prolonged hunger, the maximum score for body condition is obtained when 0% of the animals are too lean, and this happened in 41% (n = 13) of the farms (Table 6). In addition, 7% (n = 2) of the farms were classified as acceptable (between 0% and 1% of lean animals). Other 34% (n = 11) had 2–3% of too lean animals and the rest, 19% (n = 6), had more than 3% of too lean animals, the maximum being 7% in farm 32. Auditor one had less farms with problems of body condition (41%) than auditor two (75%). In terms of animals per feeder, 66% of the farms (n = 21) obtained the maximum score by having seven or fewer animals per feeder, and the others (n = 11) had more than seven animals per feeder, being eight in 9% (n = 3) and nine in 25% (n = 8). All farms had the maximum score for cleanliness of the feeders. In consequence, 31% of the farms (n = 10) obtained 100 points for this criterion and another one 80 points, so in total 34% of the farms obtained an excellent. Forty-four percent of the farms (n = 14) had more than 55 points and were classified as enhanced and 22% (n = 7) were classified as acceptable for this criterion.

In the case of the absence of prolonged thirst criterion, when animals-per-drinking-point was assessed, excellent (seven or fewer animals) was found in 34% (n = 11) of the farms (Table 6). The acceptable score (eight animals per drinker) was given to 41% (n = 13) of the farms. The rest had more than eight animals per drinker, the maximum being 13 animals per drinker in farm 32. The other two parameters had the maximum score for all farms. In consequence, 34% of the farms obtained 100 points for this criterion and another seven were as well classified as excellent for absence of prolonged thirst. The other 14 farms (44%) were classified as enhanced.

When the score of the whole principle was considered, 6% of the farms obtained 100 points and another eight (25%) were between 80 and 100 points. In consequence, 31% of the farms obtained an excellent for the principle. Another 32% (n = 11) were classified as enhanced and the other 28% (n = 9) as acceptable.

### 3.2. Good Housing

The Good Housing principle is assessed by means of the combination of three criteria: comfort around resting, thermal comfort and ease of movement.

In relation to the comfort around resting criterion, 84% of the farms were assessed with 0% of animals with sign of dirtiness, so 100 points were obtained for this measure in all cases (Table 6). The rest 16% (n = 5) showed moderate dirtiness in 1% of the assessed animals. Regarding the presence of wet animals, 88% (n = 28) of farms obtained 100 points. In farms 14, 17 and 26, the percentage of wet animals were 1.8%, 1.5% and 1.9%, respectively, so the final score was 65 points out 100, and in farm 15 the percentage of wet animals was 3%, so the final score was 0 points. For the assessment of dust, 63% of the farms were assessed with an excellent (no presence of any dust, 100 points), and the rest with an acceptable (slight evidence of dust, 70 points). For the assessment of light patterns, 69% of the farms (n = 22) had at least eight hours of light of a good quality and at least eight hours of darkness. The other farms (31%) had less than eight hours of light of a good quality per day, so 20 points were subtracted from the score of the whole criterion in these farms. In consequence, 47% of the farms (n = 15) obtained 100 points for this criterion and another five farms obtained from 80 to 90 points, so in total 63% of the farms obtained an excellent. Of the farms, 34% (n = 11) were classified as enhanced and one farm obtained an acceptable for this criterion.

In relation to the thermal comfort criterion, 75% of the farms (n = 24) had registers of environmental temperatures and they were inside the range of 1 °C to 28 °C (Table 6), and 38% (n = 12) had as well good values even when burning hair was performed, so they obtained 100 points. Another 38% (n = 12) had not registers when burning hair was performed and accordingly, in these cases 20 points were subtracted from the criterion score. Another 22% of the farms (n = 7) did not have any register of temperatures for the last few months, and one farm was outside of the range for more than two days in the last three months, so they were scored in all cases with 0 points. In consequence, overall, 75% of the farms obtained an excellent for this criterion and the rest had 0 points for the thermal comfort criterion.

**Figure 1:**
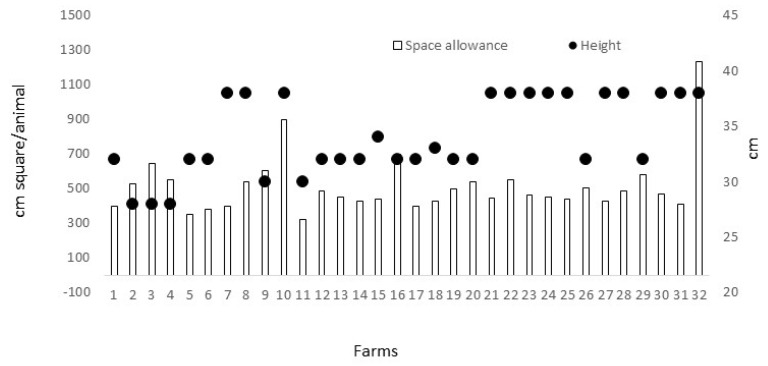
Space allowance (cm^2^ per animal) and height (cm) of the cages in growing-rabbit farms (n = 32). For space allowance, excellent is considered when at least 1500 cm^2^ are provided per animal in 90% of the cages and acceptable when this is at least 500 cm^2^ per animal. The height of the cage is considered excellent when at least 38 cm are provided in 90% of the cages and acceptable when this is at least 32 cm.

In relation to the ease of movement criterion, 41% of the farms (n = 13) had a cage height of at least 38 cm, in consequence obtaining 100 points (Figure 1). Another 44% of the farms (n = 14) had a height of between 32 cm and 34 cm, obtaining 50 points out 100. The rest of the farms, 15% (n = 5) had a cage height below 32 cm and, in consequence, 0 points were obtained. When stocking density was studied, none of the farms reached 1500 cm^2^ per animal, so none obtained an excellent for this parameter. In addition, 41% of the farms (n = 13) provided at least 500 cm^2^ per animal, obtaining 60 points out of 100 for this parameter. The maximum space allowance was given in farm 32, with 1235 cm^2^/animal (Figure 1). Fifty-nine percent of the farms were given 0 points for this parameter. Therefore, overall, for this criterion, no farms were classified with an excellent, 28% (n = 9) were classified as enhanced, with scores ranging from 55 to 75 points, 69% (n = 22) were classified as acceptable, with scores from 20 to 40 points, and one farm as not acceptable, with a final score of 0 points for the ease of movement criterion.

When the score of the whole principle was considered, none of the farms obtained 100 points, but two farms were classified with an excellent. Seventy-five percent of the farms (n = 24) were classified as enhanced, with scores ranging from 55 to 78 points. Finally, 19% of the farms were classified as acceptable, with scores ranging from 35 to 52 points for the Good Housing principle.

### 3.3. Good Health

The Good Health principle is assessed by means of the combination of three criteria: absence of injuries, absence of diseases and absence of pain induced by management.

In relation to the absence of injuries criterion, 81% of the farms (n = 26) had no animals with wounds on the ears and another two farms had up to 2% of animals with old lesions (Table 6), so 88% of the farms were classified with an excellent for this parameter. Two farms had up to 5% of the animals with ear lesions, so they were scored as acceptable and another two farms had more than 5% of animals with injured ears, with a maximum of 8% in farm 32, thus scoring 0 points. Twenty percent of the farms from observer one were affected by wounds on the ears, while 50% of the farms from auditor two. Seventy-eight percent (n = 25) of the farms had no rabbits with fallen ears and were classified as excellent. Another 16% (n = 5) of the farms had up to 1.2% of animals with fallen ears and were classified as acceptable. Finally, the other two farms had more than this 1.2% of animals affected, the maximum found being 2% of the animals with fallen ears in farms 26 and 32. Ninety-one percent (n = 29) of the farms did not have any animals with difficulties moving, and an excellent was therefore achieved. Two farms had up to 1% of the animals with moderate difficulties moving and scores as acceptable. Finally, one farm had 2% of animals with several difficulties moving and scored 0 points.

Sixty-nine percent (n = 22) of the farms had no animals with wounds on the body (Table 6) and scored 100 points. Farm 3, with 1% of animals with moderate lesions, was classified as acceptable, and the rest, with values ranging from 1% to 19% were classified as not acceptable. Seventy-eight percent (n = 25) of the farms had no animals with hairless lesions and scored 100 points. Two farms had up to 1% of animals affected and were classified as acceptable. Finally, the remaining 16% of the farms (n = 5), had more than 1% of animals affected, with the maximum being 3%, and scored 0 points for this parameter. However, while only 10% of the farms from auditor one had animals with hairless lesions, 50% of the farms from observer two had this problem. Finally, in four farms, there were cages with a risk of injuries for the animals. Therefore, 30 points were subtracted from the absence of injuries in these farms.

Overall, absence of injuries obtained 100 points in 44% of the farms (n = 14) resulting in the classification of excellent, 80 or more than 80 points, in 63% of the farms (n = 20). Of the farms, 25% (n = 8) obtained between 67 to 79 points, being classified as enhanced. Nine percent of the farms (n = 3) obtained an acceptable, between 37 and 52 points, and farm 32 received an unacceptable classification, with 0 points for absence of injuries.

In relation to the absence of diseases criterion, no animals with coughing or mange were found (Table 6). Ninety-four percent (n = 30) of the farms did not have animals seen with nasal discharge and in the other 2 farms up to 5% of the animals were found affected, and they were scored as acceptable for this parameter. Ninety-one percent (n = 29) of the farms did not have problems with ocular discharge, two farms had up to 1% of their animals affected and scored as acceptable and another one had more than 2% of the animals affected and scored 0 points. A total of 69% of the farms had no problems of skin condition (dermatophytosis, dermatitis or abscesses), while the rest 31% of the farms (n = 10) were affected with dermatophytosis and, accordingly, scored 0 points. However, while only a 20% of the farms assessed by auditor one were affected, auditor two found this problem in 66% of the farms. Eighty-one percent of the farms (n = 26) had no animals with neck torsions and scored 100 points. However, the other 19% of the farms had between 1% and 2% of the animals affected with moderate cases of neck torsion and scored 0 points for this parameter. Again, auditor one found this problem only in one farm (5%), while auditor two in five (42%). Sixty-nine percent of the farms did not have any cases of animals sneezing, while the rest—31%— had at least one. Nevertheless, three farms had just one cage with problems, so they were scored as excellent. Two other farms had cases in two cages, and scored as acceptable, and another five farms had more than two cages affected and scored 0 points. Auditor one registered animals sneezing in just one farm, while for auditor two, 75% of the farms were affected with this problem.

**Figure 2:**
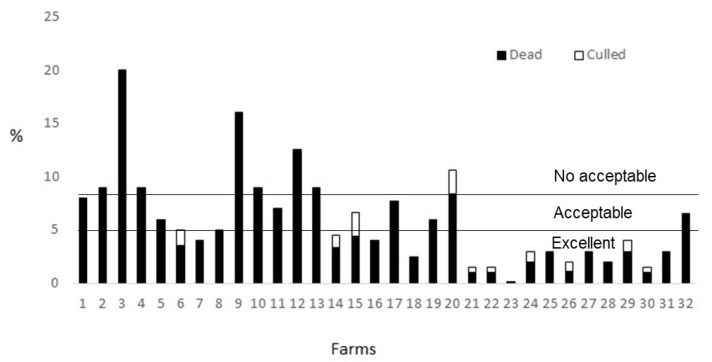
Mortality rates (in white: culled animals; in black: dead animals or overall mortality rates when % of culled animals were not given by the farmer) in growing-rabbit farms (n = 32) in relation to the classification of the animal welfare protocol, where up to 5% of mortality in at least the last thee months is considered excellent, up to 8% of mortality is considered acceptable, and any other value as not acceptable.

A total of 53% of the farms (n = 17) obtained an excellent for mortality (Figure 2), and another 22% (n = 7) were classified as acceptable. The rest of the farms had a “not acceptable” score, the highest mortality recorded being 20% in farm 3. Overall, the mortality rates registered in farms from auditor one were higher than those from auditor two. Only 34% of all the farms (n = 11) registered the culling percentage in relation to the total mortality rates. In two farms, culling represented more than 40% of mortality, and was classified as excellent. In another 28% (n = 9), culling represented from 20% to 40% of mortality, and was classified as acceptable. For the rest of the farms, 66% (n = 21) culling either accounted for 0% of mortality, or the information was not provided and thus they were given 0 points. A total of 10 farms were found to have problems with cleanliness. Twenty-eight percent (n = 9) of the farms were found with up to five cages that were partly dirty, and so ten points were subtracted from the absence of disease criterion for these farms. One farm had more than five cages partly dirty, and so 20 points were subtracted from the whole criterion score in this case. In fact, auditor one reported no problems of cleanliness in their farms, while auditor two reported that ten of the 12 farms assessed had a problem of dirtiness. Finally, in five of the 32 farms (16%) flies and their eggs were found in the facilities and, as a consequence, 20 points were deducted from the whole criterion in these cases.

In total, none of the farms obtained 100 points for the absence of disease criterion, and 28% (n = 9) obtained an excellent (scores ranging from 82 to 97). In addition, 47% of the farms (n = 15) were classified as enhanced (scores ranging from 62 to 77) and the rest, 25% (n = 8), were classified as acceptable (scores ranging from 29 to 54 points) for the absence of disease criterion.

In relation to the absence of pain induced by management criterion, only one of the farms performed emergency killing with any of the methods considered in the protocol (Table 6). The farmers not using any of these systems declared that they used just cervical dislocation or blunt force on the head for the animals. Four farmers declared indeed not to use any system for emergency killing. In consequence, one farm obtained an excellent for this criterion and the rest received a “not acceptable” score.

When the score for the whole principle was considered, none of the farms obtained an excellent. Eighty-one percent of the farms (n = 26) obtained an enhanced qualification for this principle, ranging from 55 to 79 points, 16% (n = 5) had acceptable classification (scores ranging from 36 to 49 points) and one farm (32) had a “not acceptable” classification, with a score of 12 points.

### 3.4. Appropriate Behaviour

The Appropriate Behaviour principle is assessed by means of the combination of three criteria: social behaviour, other behaviours and the human–animal relationship.

In relation to the social behavior criterion, no animals biting other animals were found in any of the cages assessed in the 32 farms (Table 6). In addition, no cases of isolated animals (animals with no visual contact with other animals) were found. In consequence, the 100 points for this criterion was achieved by all of the farms. In relation to the other behaviours criterion, no presence of animals showing abnormal behaviours, such as biting elements of the cage or stereotypies, were found in 97% of the farms (n = 31). In consequence, all farms except one (farm 32) obtained 100 points for this criterion. In farm 32 it was observed how animals were scratching or biting the cage in three cages, and as a consequence, it scored 0. In relation to the human–animal relationship criterion, 9% of the farms (n = 3) had all personnel trained in animal welfare and obtained 100 points. In another 6% of the farms (n = 2), at least one of the farmers had a specific training course in animal welfare, but not all of them, in this case achieving 50 points, an acceptable score for the criterion. Finally, the rest farms, 84% (n = 27) obtained 0 points for not having anyone with a specific training course, so the criterion was classed as not acceptable in these cases.

When the score for the whole principle was considered, 9% (n = 3) obtained 100 points and another 6% (n = 2) a score from 80 to 100, so 15% of the farms were assessed as excellent for this principle. Eighty-one percent of the farms (n = 26) were assessed with 70 points and, in consequence, classified as enhanced, and one farm (32), had an acceptable classification, with 35 points, for the appropriate behaviour principle.

### 3.5. Overall Assessment

**Figure 3:**
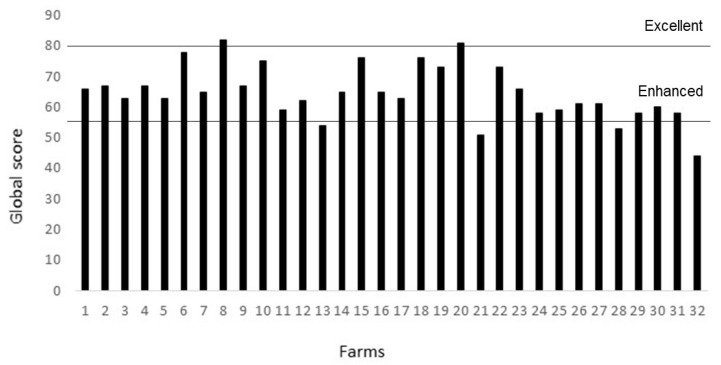
Overall welfare assessment scores, out of a possible 100, for 32 growing-rabbit farms located in Spain and Portugal.

Considering the overall score for all of the farms, where good feeding provides 15%, good housing 30%, good health 35% and appropriate behaviour 20% of the final score, only 6% of the farms (n = 2) were classified with an excellent, farm 8 and 20, with 82 and 81 points, respectively (Figure 3). Eighty-one percent of the farms were classified as enhanced, with values ranging from 58 to 78 points. Finally, 13% of the farms (n = 4) were classified as acceptable, with values ranging from 44 to 53 points.

## 4. Discussion

### 4.1. Good Feeding

Inside the Good Feeding principle, only one animal-based measure is used: body condition. Sixty percent of the farms assessed had at least one animal that was too lean, and more than 50% of the farms had more than 1% of the animals that were too lean (with the maximum reaching a 6% and 7% in one farm according to each auditor), so this was the most frequent problem seen in the farms in terms of animal based measures (Table 6). Although farms from auditor one were less affected (41%) than farms from auditor two (75%), diseases may also affect body condition [9], so the differences found between auditors in the health status of the farms could explain it. Although in other species, such as poultry, pigs and cattle, diseases also affect body condition, the measure is considered reliable for the assessment of prolonged hunger [6,8,10]. It is stated that one feeder per 3–4 meat rabbits should be sufficient [9]. But under commercial conditions even 8–10 rabbits can be held per feeder without causing any problems [4]. In the present study, 67% of the farms had seven or less animals per feeder (and 34% up to 4 animals), while a 25% arrived to nine. However, it is not clear any relationship between these numbers and the presence of lean animals in the farms studied, so any refinement of the protocol at this respect need of further studies.

Water plays an important role in the digestive process. Rabbits with an insufficient supply of water will limit their feed intake [11]. In fact, rabbits have great water requirements and consume approximately twice as much water as feed, but there are no animal-based indicators available to measure prolonged thirst in a farm by visual inspection. For, other species, no animal-based parameters are currently in use for this criterion and, alternatively, the number of drinking points and sometimes also the cleanliness and functioning of drinkers is assessed [6,8,10]. According to Verga et al. (2006) [12], the minimum provided should be one nipple for every 10 rabbits. In the present study, 75% of the farms provided one drinker for every eight animals and, only in punctual cases such as Farms 11 and 32, with 12 and 13 animals, respectively, had more than 10 animals per drinker. In any case, rabbits must have continuous access to safe drinking water and, for this, it is important that nipples are clean, e.g., no hairs visible, no green rash, and are working perfectly. In this case, all farms assessed obtained the maximum score because of the cleanliness of the drinkers.

In contrast to other protocols [6,8,10], in the present one more weight is given to the absence of prolonged hunger (65%) than to that of prolonged thirst (35%). This was the result of long discussions between experts. Although everybody agreed that thirst is worse than hunger, it was considered that when assessing body condition, you are closer to assessing chronic hunger than you are to thirst when you are assessing the number of animals per drinker, which is just assessing competition for resources. This is an important point to take into account as, in fact, although the name of the criterion is absence of prolonged thirst, the indicator used in this protocol is not really assessing this, as the mental experience of thirst cannot be assessed. This limitation should also be considered for the overall protocol for reflecting the holistic welfare state. Nevertheless, if in the future a good animal-based parameter is validated and feasible for the assessment of thirst in a growing-rabbit farm, the weights of absence of prolonged hunger and absence of prolonged thirst should be reconsidered in the present protocol. For instance, recently Kells et al., (in press) [13] validated a skin test for the assessment of dehydration in healthy calves of 4–5 days old deprived of feed and water for 24 h. Although the species and the context are not comparable, these types of approaches could be of interest for further improvements of the protocol.

### 4.2. Good Housing

Inside the good housing principle, three main aspects should be considered: comfort around resting, thermal comfort and ease of movement. Two animal-based measures related to comfort around resting are wet and dirty animals. In the present study, only in three farms were wet animals found, and they represented less than 2% of the animals assessed, so this is not a frequent problem. In the case of dirtiness, something similar is happening, as, when found, only moderate cases were described in up to 1% of the animals affected. The respiratory tract of rabbits is irritated by fine dust in the air [14], so dust levels should not be too high. Nonetheless, there is no literature showing which dust levels are acceptable or not. For other species, Welfare Quality applies a dust-sheet test, which is a simple procedure indicating the amount of dust in the air [8]. In this case, 63% of the farms had excellent results with this test, but the rest 37% had some rests of dust in the test. Although, in general, the farms with these conditions had lower scores for nasal and ocular discharge, at the moment, it is suggested to maintain both indicators in an assessment protocol. Finally, light quality is another important aspect to assess when comfort around resting is considered. Lighting should provide uniform illumination and permit the effective observation of rabbits. Continuous lighting (i.e., no dark period in a 24-h cycle) negatively impacts welfare and health, and a natural light–dark pattern enables the rabbit to apply its natural rest–activity rhythm [11]. In this case, 31% of the farms did not provide enough light to the animals, so this is a critical point to consider.

The second criterion to consider inside good housing, is thermal comfort. The results show that most of the farmers (75%) have registers of temperature, not arriving to the maximum values established in the protocol. In addition, 50% were aware of the importance of considering the risk that hair burning has in achieving a high environmental temperature, locally, at specific areas inside the building. The range of temperatures proposed by the experts for growing-rabbits is a maximum of 28 °C, and although thermal stress in rabbits has been defined at 30 °C [15,16], temperatures from 25 °C to 30 °C should be considered already as slightly or moderately stressful for these animals [17]. In fact, Dalmau et al., 2015 [17], described an increase in faecal cortisol metabolites and behavioural changes in rabbits subjected to mean temperatures of 27 °C for six hours a day, when compared to animals subjected to 20 °C. Therefore, in the future, the protocol should reconsider this temperature to be closer to the 15–20 °C defined as ideal by EFSA (European Food Safety Authority) (2005) [9]. Another even better option, is to consider not just the temperature, but also the temperature and humidity by means of an index combining both factors, such as the Temperature–humidity index (THI) discussed by Marai et al. (2002) [18]. According to these authors, absence of thermal stress is considered when the THI is below 27.8, as moderate heat stress when it ranges from 27.8 to 28.9, as severe heat stress when it ranges from 28.9 to 30.0 and as very severe stress when it is above 30.0. Nevertheless, if nowadays 25% of the farmers assessed did not have registers, to include the need for humidity assessment could dramatically reduce the number of farms with available data for the assessment, so this change should be considered as well in terms of feasibility. On the other hand, although the experts considered that rabbits are well adapted to temperatures below 10 °C, any future improvement of the protocol should consider moving the low value of the range allowed (1–28 °C) closer to the optimal temperatures of 15–20 °C as well [9].

Ease of movement considers the height of the cage and space allowance. The height of the cage is important because rabbits inspect their environment by showing ”standing up” behaviour [9]. According to EFSA (2005) [9], fattening rabbits should be kept in collective cages with a minimum 75–80 cm depth, 35–40 cm width and 38–40 cm height. In the present study, only 41% of the farms provided at least 38 cm height to the animals and a 15% were below 32 cm, so this is a critical point to be considered. Fattening rabbits in medium-sized groups (7–10 rabbits) is common practice in all commercial rabbit-producing countries except Italy and Hungary, where rabbits are usually kept two per cage from weaning until slaughter. The higher slaughter age (80–90 days) necessary to reach the high market weight requested by consumers (2.5–2.6 kg on average) compared to France (2.3–2.4 kg) and Spain or Portugal (1.8–2.2 kg), and the consequent possibility of increased aggressive behaviour and wounds are the two main reasons for this housing system [19]. There are several studies, with respect to stocking density for meat rabbits, and, in general, a maximum stocking density of 16 rabbits/m^2^ or 40 kg/m^2^ is seen as acceptable in terms of welfare [20,21]. The 500 cm^2^ per animal considered as acceptable in the present protocol is exactly 40kg/m^2^ for an animal of 2 kg body weight. If the final slaughter weight was 1.8 kg, the space allowance would be 450 cm^2^ per animal, and at 2.2 kg would be 550 cm^2^ per animal. Therefore, a change of the parameter to kg/m^2^ should be considered in the future for the protocol. In any case, according to the expert consultation performed in EFSA (2020) [22], restriction of movement was the main welfare problem identified in growing-rabbits, so a possible improvement could be to increase the weight of this parameter in the final score of the protocol (now it is 15 points out of 100).

### 4.3. Good Health

This principle includes the absence of injuries, diseases and pain induced by management. Wounds on the body were seen in 50% of the assessed farms, being the most common problem inside the absence of injuries criterion, while fallen ears and lameness were seen in few animals. Wounds can be caused by inadequate equipment (e.g., sharp parts of cages), or by mutilative or aggressive behaviour of other rabbits [23]. In the present protocol, only lesions of 2 cm or bigger were considered. This is an important point to take into account, because according to Rauterberg et al. (2019) [24], rabbits have a high prevalence of lesions of less than 1 cm, especially in the area of the ears. However, these small lesions are difficult to assess in commercial conditions due to the light conditions of some farms and the angle of vision of the animals in the cages. As the present protocol avoids catching the animals in order to reduce the stress associated with human contact, to include these small lesions without catching the rabbits dramatically reduced the inter-observer repeatability of this parameter, so, as decided in the Welfare Quality for piglets [6], only lesions clearly visible for any auditor (at least 2 cm in size) are considered. This means that although the assessment of wounds showed a good capacity of discrimination between farms, the protocol does not consider all the wounds that a rabbit could have, but rather only a portion. Hairless lesions and wounds on the ears were found in just 10% and 20%, respectively, of the farms assessed by auditor one and in 50% of the farms from auditor two. Although problems of reliability should not be discarded for these two measures, differences in the health status of the farms assessed by the two auditors could explain these differences. In fact, auditor two reported a high incidence of signs compatible with dermatophytosis in the farms assessed, as most of the lesions found were circular with erythema, with a rough and dry surface [21], compatible with this explanation.

Accordingly, auditor two found more health problems in their farms than auditor one. These differences were focused in the incidence of skin condition problems, such as dermatophytosis, with just 20% of the farms from auditor one and 66% of the farms from auditor two affected, and respiratory problems, with few observed in farms one to 20, but with a high incidence in farms from 21 to 32 (Table 6). For instance, auditor one registered animals sneezing in just one farm, while for auditor two 75% of the farms were affected with this problem. Skin alterations and respiratory problems are easier to train for auditors than other parameters such as lameness, that needs more exercises to ensure good repeatability between auditors. In rabbits, it is easy to check the eyes and nose for alterations, and sneezing, when focused on a cage, is also very easily assessed, with a high agreement between auditors during trainings. Therefore, although it is not possible to discard an auditor effect, other reasons would better explain the differences found. For instance, it is described that dermatophytosis appears in environments with a high relative humidity, temperature changes, contact with contaminated soil and poor hygiene [25]. Auditor one reported no problems of cleanliness in their farms, while auditor two reported that ten of the 12 farms assessed had a problem of dirtiness. Again, three reasons could be argued for these differences. (1) A different perception of what is dirty or not by part of the auditors. (2) The fact that most of the farms from auditor one were in a regimen of all in all out. This means that growing-rabbits were moved to specific cages for these animals and after finishing the growing period, cages were sanitated. In the farms from auditor two, growing-rabbits were maintained in the doe’s cage after weaning (mothers were moved) and does at different stages of production were maintained in the same building, impairing sanitation. (3) Another reason could be the fact that farms from auditor one were inside an animal welfare certification scheme and farms from auditor two were not. In any case, another frequent disease problem in rabbit farms associated with poor hygiene is Pasteurellosis [18,26]. Signs of Pasteurellosis can include discharge (from the eyes and nose), matted forepaws, sneezing, respiratory distress, abscesses and neck torsion [18]. Only in one farm from auditor one, but in five from auditor two (42%) was a problem of neck torsion found. This, again, could be an auditor effect, but the most suitable explanation is a higher incidence of health problems (skin condition and respiratory disorders) related with sanitation in farms from auditor two.

The mortality rates in farms from auditor two were far lower than those found from auditor one. As this information is based on registers, no auditor effect is expected, suggesting that most of the differences found between both auditors in the health measures were due to a different management in the farms selected. Farms from auditor one had few health problems the day of the audit but had high percentages of dead animals, and farms from auditor two had more health problems the day of the audit but lower percentages of dead animals. Of course, the ideal situation is those in which the health problems and the mortality and culling rates are both low, as occurred in farm 18. In any case, an assessment protocol should penalize both situations, when a high incidence of animals with health problems is present, and when a high percentage of dead animals (even when culled) are found. Nowadays, inside the absence of disease criterion, dead and culled animals have a weight of 30% of the total score, so if a high percentage of the animals are dead, as is the case in farm 3, there is a risk of having a good overall score for absence of disease due to the lack of problems in the rest of the measures. For this reason, going forward, the protocol should address how to penalize the overall score when this is happening to avoid this compensation between measures. For instance, by minusing 10 points from the overall score when the mortality is higher than 8%, 20 points when it is higher than 15% and 30 points if higher than 20%. However, as done in previous stages, this should be done after expert consultation and, if possible, after having a high number of farms (around 100) assessed with the protocol. On the other hand, only 31% of the farmers provided the percentage of culled animals. As this is critical information, the protocol should consider this as a red line and consider an overall score of 0 points if the farmers have not registered this information.

According to EFSA (2020) [22], digestive troubles are probably one of the main hazards for the welfare of rabbits. This can range from slight troubles (transitory low feed intake, light diarrhoea) to acute, painful ones (no feed intake, weight loss, acute diarrhoea or ceacal impaction, intestinal inflammation, gastric or intestinal dilatation or swelling, mucus excretion, etc.) [22]. Although these problems usually affect young rabbits around the period of weaning, later, during the growing period, digestive disorders are the main cause of mortality [22]. A possible clinical sign of digestive disorders in rabbits, in addition to body condition, is the presence of liquid manure around the anus, or a firm abdomen. To assess this, the animals need to be caught and turned over to check the anus and palpate the abdomen. This was carried out in a previous version of the protocol, but even in farms with known problems of enteropathies and high mortality rates due to this cause, the percentage of animals found with clinical signs during the assessment was very low, as in most of the cases the animal dies very quickly after the appearance of clinical signs. In addition, while does and bucks are used to being handled, growing-rabbits are not, so management during the assessment by itself is more dangerous for the welfare of the animals due to the stress caused by the auditor. As a consequence, while in the protocol developed for does and bucks this parameter was maintained [27], for growing-rabbits it was considered that the information obtained by checking clinical signs of diarrhoea and caecal impaction (in addition to mortality rates and body condition, already assessed), does not compensate for the stress produced to obtain the data.

Pain induced by management includes how emergency killing is performed. Most of the farmers declared to use just cervical dislocation as a system for emergency killing. However, according to the recommendations of EFSA (2020) [28], cervical dislocation is not considered a humane killing method and therefore it should be only applied on unconscious animals. In addition, the hazards related to cervical dislocation include “manual restraint” (leading to pain and fear) and “incorrect application” (leading to the absence of unconsciousness, pain, fear and distress) [28]. For these reasons, in the present protocol, this system is not considered as correct. Certainly, this is one critical point that needs to be improved in the rabbit farms assessed, and there is probably a need for good guidelines to be provided by producer associations and supervised by researchers.

### 4.4. Appropriate Behaviour

The Appropriate Behaviour principle is assessed by means of the combination of three criteria: social behaviour, other behaviours and the human–animal relationship. Ear damage, scratches and wounds are indicators of negative social behaviour (i.e., aggressiveness), but they have other causes as well (see absence of injuries above), so the observation directly on the animals of this behaviour could be a better indicator. The age of growing-rabbits is an important factor, as rabbits at the end of the growing period may show more aggressive behaviour (due to maturation of the rabbits) [20,29,30]. This can be easily seen in production systems raising heavier animals than those produced in Spain or Portugal, which could explain the lack of negative social behaviour found in the present study. Another explanation is the fact that all growing-rabbits were fed ad libitum, as Dalmau et al., 2015 [31] described that when fed in a restricted manner, growing-rabbits performed eight to 12-fold more agonistic behaviours than when fed ad libitum. Nevertheless, another explanation could be an insufficient capacity of discrimination of the parameter among the assessed farms, for instance due to insufficient time of observation (just two minutes per cage) or the number of cages assessed (just 10 per farm for this parameter). Therefore, a test of the same parameter in older animals and/or restricted feeding would be necessary to ascertain if the results found in the present study were due to the lack of aggressiveness of the animals in all farms, or a problem with the sensitivity of the parameter. The results showed that none of the animals present in the farms assessed were isolated.

Both in growing and adult animals, stereotypies, that is, abnormal behaviour repeated obsessively without any apparent aim, have often been described [32,33]. Stereotypies and abnormal behaviour are indicators of reduced welfare in rabbits. These behaviours can be: head shaking, swaying, wire gnawing, wall pawing and over-grooming [9,29,34,35]. In the present study, only one farm with presence of these abnormal behaviours, 32, was found (with 13% of the animals affected). In fact, this farm was the worst, in general, for several measures. Therefore, as discussed previously for negative social behaviour, and although the methodology (two minutes of observation per cage) was tested previously in other farms with positive results (presence of abnormal behaviours identified, as what happened in farm 32) and the time dedicated to assess abnormal behaviours is twice than that used in other species, such as pigs, for the same purpose [6], it cannot be discarded that some adjustment to the methodology might be necessary to increase its sensitivity. In addition, several authors have reported the importance of enrichment for growing-rabbits [36,37], so its presence or absence should also be considered in a future version of the protocol, since it is already considered in the protocol for does and bucks [27].

A good human–animal relationship promotes rabbit welfare. With proper handling, rabbits experience less stress and fear, and the risk of injury to the animals and handlers is greatly reduced [9]. Management practices have a significant impact on animal health, welfare, and productivity [38], and the training of farmers has a great impact on this. In the present protocol, an excellent is considered when all the personnel in contact with the animals have specific training in animal welfare, but this only occurred in three farms, with another two farms having at least one of the persons trained. Therefore, this is another key point to improve in rabbit farms in the near future.

### 4.5. Overall Assessment

Values ranged from 44 to 82 points in the 32 farms, so from the worst to the best farm there were 38 points of difference. The lack of more farms in the excellent category, and the fact that none obtained 100 points, would show that there are possibilities for improving welfare in all farms assessed. The fact that the minimum value obtained was 44 could be due to a lack of sensitivity of the protocol in the lower levels, or to the natural bias in the selection of the farms assessed. In fact, the farms from auditor one were of farmers interested in being certified in animal welfare, with previous knowledge of the protocol (although they were never audited previously with this tool) and advised that they needed to achieve at least 55 points, so it is easy to understand that just one, with a total score of 54 points, remained out of this condition after the assessment. In fact, this could show that the protocol is easy to understand for farmers and they can apply self-assessments before being audited by external assessors. In the case of farms from auditor two, they were not in a certification program, but farmers voluntarily offered for their farms to be visited and audited, so it can be expected that these farms could be positively biased in relation to the global population of farms.

Altogether, with proper training, the protocol seems reliable for the assessment of animal welfare. However, for the criteria social behaviour and other behaviours, further research is needed to ascertain if the methodology and times of observation used are enough to ensure the absence of problems or need to be changed. Auditor one used a mean of 300 animals per farm per clinal measure, while auditor two used a mean of 170 animals per farm. Sample sizes need to find a balance between the reliability and feasibility of a protocol. In the Welfare Quality protocols, this balance was found in using 100 laying hens, 150 broilers and a maximum of 150 pigs for clinical measures [6,7], less than the animals used in the present study for rabbits. In terms of reliability, although sampling 10%, 12% and 33% of the population (farm, 1, 13 and 16, respectively) is always more representative than sampling 2% of the population (farms 5, 6, 14 and 18), the results found under both scenarios were similar (Table 6). Nevertheless, this limitation should be considered for the protocol in farms with more than 10,000 growing-rabbits.

In terms of feasibility, the objective of the protocol was to fix a maximum time of 3 h to take all the data. This is because a second protocol to assess the welfare of does, bucks and kits was developed as well [27]. As most of the farms have does with kits and growing-rabbits, the objective is to apply both protocols in a journey (maximum of seven hours). Nowadays, with 20 to 50 cages and around 170–300 rabbits assessed for clinical measures, the assessment takes from two to two and a half hours, so there is still a chance to increase the time dedicated to assess social and other behaviours, if needed.

## 5. Conclusions

Overall, the protocol shows a good capacity for discrimination between farms, so it can be a useful tool. However, as mentioned above, a system for avoiding compensation between measures, at least for the most critical ones, such as mortality rates, should be incorporated by means of expert consultation and once enough data is available. The good feeding and appropriate behaviour principles, obtained the most homogeneous results from both auditors, while the good housing and good health principles showed the most variation between both. In the case of good housing the differences were due to the facilities, and it is discarded that there was a potential effect of the auditor. The case of good health is due to animal based measures, and specially to skin condition and signs of respiratory disorders, but the presence of dermatophytosis and Pasteurellosis in some of the farms assessed, instead of an auditor effect, is considered the most suitable explanation. The main critical points to be solved in the growing-rabbit farms were to provide more space to the animals, accurately register the number of animals culled from the total mortality rates, change cervical dislocation for another emergency killing method and provide training in animal welfare to the farmers. In some cases, it is important to register maximum and minimum daily temperatures, to provide at least eight hours of sufficient light per day and to improve sanitation.

## Figures and Tables

**Table 1:** Principles and criteria defined in the European Welfare Quality project to assess animal welfare [3].

Principles	Criteria
Good Feeding	Absence of prolonged hunger
	Absence of prolonged thirst
Good Housing	Comfort around resting
	Thermal comfort
	Ease of movement
Good Health	Absence of injuries
	Absence of diseases
	Absence of pain induced by management
Appropriate Behaviour	Social behaviour
	Other behaviours
	Human–animal relationship
	Positive emotional state

**Table 2:** Parameters used to assess the absence of prolonged hunger and the absence of prolonged thirst criteria within the Good Feeding Principle. The weight means which percentage of the score of the total criterion is represented by each parameter. Each parameter can be assessed according to two categories, excellent and acceptable, and are scored accordingly.

Criterion	Weight	Parameter	Weight	Categories	Definition of Categories	Score
Absence of prolonged hunger	65% of the Good Feeding Principle	Body condition	60%	Excellent	0% of lean animals	100
				Acceptable	Up to 1% of lean animals	65
		Animals per feeder	25%	Excellent	Up to 7 animals per feeder	100
				Acceptable	Up to 8 animals per feeder	60
		Cleanliness of feeders	15%	Excellent	95% clean and 5% partly clean feeders	100
				Acceptable	80% clean and 20% partly clean feeders	45
Absence of prolonged thirst	35% of the Good Feeding Principle	Animals per drinker	40%	Excellent	Up to 7 animals per drinker	100
				Acceptable	Up to 8 animals per drinker	85
		Working drinkers	35%	Excellent	100% with good water flow	100
				Acceptable	90% with good water flow	55
		Cleanliness of drinkers	25%	Excellent	100% of clean drinkers	100
				Acceptable	90% of clean drinkers	60

**Table 3:** Parameters used to assess the criteria of comfort around resting, thermal comfort and ease of movement within the Good Housing Principle. The weight means which percentage of the score of the total criterion is represented by each parameter. Some parameters, such as light quality, are only considered in the score when the acceptable value is not achieved, subtracting points from the overall score. Each parameter can be assessed according to two categories and are scored accordingly.

Criterion	Weight	Parameter	Weight	Categories	Definition of Categories	Score
Comfort around resting	35% of the Good Housing Principle	Wet animals	30	Excellent	Less than 1% of animals wet	100
				Acceptable	Less than 2% of animals wet	65
		Dirty animals	60	Excellent	Up to 0.5% moderately dirty, 0% severe	100
				Acceptable	Up to 1% moderate and 0.5% severe	65
		Dust	10	Excellent	No dust presence	100
				Acceptable	Minimal dust present	70
		Light quality	0 (−20)	Acceptable	8 h of light and 8 h of darkness and enough light to check animals	0
				Not acceptable	Any other situation	−20
Thermal comfort	15% of the Good Housing Principle	Temperature	100	Excellent	Last 3 months with range of 1 °C to 28 °C	100
				Acceptable	Up to two days outside of this range	50
		Burning hair	0 (−20)	Acceptable	During burning hair not >28 °C	0
				Not acceptable	Not registered or >28 °C	−20
Ease of movement	50% of the Good Housing Principle	Height of the cage	40	Excellent	38 cm at least in 90% of the cages	100
				Acceptable	32 cm at least in 90% of the cages	50
		Stocking density	60	Excellent	1500 cm^2^ per animal in 90% of cages	100
				Acceptable	500 cm^2^ per animal in 90% of cages	60

**Table 4:** Parameters used to assess the absence of injuries, absence of diseases and pain induced by management procedures criteria within the Good Health Principle. The weight means which percentage of the score of the total criterion is represented by each parameter. Some parameters, such as risk of injuries, are only considered in the score when the acceptable value is not achieved, subtracting points from the overall score. Each parameter can be assessed according to two categories and are scored accordingly. In some cases, the category Not acceptable can have more than one score.

Criterion	Weight	Parameter	Weight	Categories	Definition of Categories	Score
Absence of injuries	40% of the Good Health Principle	Wounds on the body	30	Excellent	Up to 0.4% moderate and 0% severe	100
				Acceptable	Up to 1% moderate and 0.4% severe	50
		Wounds on the ears	20	Excellent	Up to 2% old and 0% fresh lesions	100
				Acceptable	Up to 5% old and 1% fresh lesions	60
		Fallen ears	10	Excellent	Up to 0.6% with fallen ears	100
				Acceptable	Up to 1.2% with fallen ears	70
		Hairless areas	10	Excellent	Up to 0.2% with hairless areas	100
				Acceptable	Up to 1% with hairless areas	70
		Gait score	10	Excellent	Up to 0.4% moderate and 0% severe	100
				Acceptable	Up to 1% moderate and 0.4% severe	65
		Risk of injuries	0 (−30)	Acceptable	No cages with risk of injuries	0
				Not acceptable	1 cage with risk of injuries	−15
				Not acceptable	More than 1 cage with risk of injuries	−30
Absence of diseases	40% of the Good Health Principle	Mortality	20	Excellent	Up to 5% in at least the last 3 months	100
				Acceptable	Up to 8% in at least the last 3 months	60
		Culling	10	Excellent	At least 40% of the mortality rate	100
				Acceptable	At least 20% of the mortality rate	70
		Coughing	10	Excellent	1 cage with less than 3 events in 2 min	100
				Acceptable	2 cages with <3 and 1 cage with 3	70
		Sneezing	10	Excellent	1 cage with less than 3 events in 2 min	100
				Acceptable	2 cages with <3 and 1 cage with 3	70
		Nasal discharge	10	Excellent	Up to 1% of animals affected	100
				Acceptable	Up to 5% of animals affected	70
		Ocular discharge	10	Excellent	Up to 0.8% of animals affected	100
				Acceptable	Up to 2% of animals affected	70
		Dermatophytosis, dermatitis, abscesses	10	Excellent	0% of skin problems	100
				Acceptable	0% of animals with dermatophytosisUp to 0.5% of animals with dermatitis or abscesses	70
		Neck torsions	20	Excellent	Up to 0.2% moderate, 0% severe	100
				Acceptable	Up to 0.5% moderate, 0.2% severe	60
		Mange	0 (−20)	Acceptable	0% of animals affected	0
				Not accept.	At least one animal affected	−20
		Cleanliness of facilities	0 (−20)	Acceptable	Up 2 cages partly dirty	0
				Not acceptable	Up 5 cages partly dirty and up to 2 cages dirty	−10
				Not acceptable	Any other case	−20
		Flies presence	0 (−20)	Acceptable	No flies nor fly eggs present	0
				Not acceptable	Flies or fly eggs present	−10
				Not acceptable	Flies and fly eggs present	−20
Absence of pain induced by management	20% of the Good Health Principle	Killing methods	100	Excellent	Penetrative captive bolt with pithing, penetrative captive bolt with bleeding, penetrative captive bolt with neck dislocation, electronarcosis with neck dislocation, electronarcosis with bleeding and lethal injection	100
				Not acceptable	None of the previous systems	0

**Table 5:** Parameters used to assess the social behaviour, other behaviours and human–animal relationship criteria within the Appropriate Behaviour Principle. Each criterion is assessed by a single measure, except social behaviour. In this case, if no animals isolated are found the score is not altered, but if any are found then some points can be subtracted from the whole criterion. In some cases, the category Not acceptable can have more than one score.

Criterion	Weight	Parameter	Weight	Categories	Definition of Categories	Score
Social behaviour	35% of the Appropriate Behaviour Principle	Negative social behaviour	100	Excellent	No animals biting other animals	100
				Enhanced	One animal biting another animal	70
				Acceptable	Two animals biting another animal	40
				Not acceptable	Three animals biting another animal	10
				Not acceptable	More than three animals biting another animal	0
		Isolated animals	0 (−100)	Acceptable	No animals visually isolated	0
				Not acceptable	Up to 10% of animals isolated	−45
				Not acceptable	More than 10% of animals isolated	−100
Other behaviours	35% of the Appropriate Behaviour Principle	Abnormal behaviours	100	Excellent	0% with abnormal behaviour	100
				Acceptable	Up to 2 cages with animals found with abnormal behaviour	55
Human-animal relationship	30% of the Appropriate Behaviour Principle	Training of personnel	100	Excellent	All personnel in contact with animals trained in animal welfare	100
				Acceptable	At least one person trained in animal welfare	50

**Table 6:** Sampling used (% of the animals assessed out of the total present in the farm) and the results found for different parameters in farms 1 to 32 of growing-rabbits reared for meat production. (%) is the percentage of animals affected out of all the assessed animals. 0 means excellent situation, 1, acceptable situation (moderate problems) and 2, not acceptable situation (severe problems). (n) is the number of cages affected from a total of 20 to 50 cages assessed.

Farm	1	2	3	4	5	6	7	8	9	10	11	12	13	14	15	16	17	18	19	20	21	22	23	24	25	26	27	28	29	30	31	32
Sampling (%)	10	4	3	5	2	2	3	5	4	3	4	9	12	2	3	33	6	2	4	8	4	4	4	3	3	3	3	2	2	5	3	3
Lean animals (%)	0.6	6	6	1.1	0	0	0	0	2	1.5	1.7	2.5	1.4	2.7	0.7	0	3.6	0	0	2.1	2.8	0	1	0	0	2	1	2	2	6	2	7
Animals per feeder	5	3	2	2	4	5	5	6	3	2	5	6	5	4	4	3	4	4	4	7	9	7	9	9	9	8	9	8	8	9	10	5
Animals per drinker	10	5	4	4	8	9	5	6	5	2	12	6	9	8	8	3	8	8	8	7	9	7	9	9	9	8	9	8	8	9	10	5
Wet animals (%)	0	0	0	0	0	0	0	0	0.9	0	0	0	0	0	0.7	0	1.4	0	0	0.6	0	0	0	0	0	1	0	0	0	0	0	0
Dirty animals (%)	0	0	0	0	0	0	0	0	0	0	0	0.6	0.6	0.3	4	0	0	0	0	0	0	0	0	1	1	2	0	0	0	3	0	1
Dust (0–1–2)	0	0	0	0	0	0	0	0	0	0	0	0	0	0	0	0	0	0	0	0	1	1	1	1	1	1	1	1	1	1	1	2
Light quality (0–2)	0	0	2	0	0	0	0	0	0	0	0	0	0	0	0	2	0	0	0	0	2	2	2	2	2	2	0	0	0	0	2	2
Temperature (0–2)	2	0	0	0	2	0	2	0	0	2	2	0	2	0	0	2	0	0	2	0	0	0	0	0	0	0	0	0	0	0	0	0
Wounds body (%)	0	0.8	1	1.1	0	0	0	0	0	0.5	0	0	0.6	1.2	0.3	0	0	0	0	0.3	4	0	0	6	8	0	1	1	3	0	5	19
Wounds ears (%)	0	0	0	0	0	0	0	0	0	0	0.7	0	0	0.3	3	0	0	0.4	0	0	0	0	0	8	5	5	1	0	0	0	2	8
Fallen ears (%)	0	0	0	0	0	0	0	0	0	0	0	0	0	0	0	0	0	0	0	0	0	1	0	1	0	2	0	1	0	1	1	2
Hairless areas (%)	0	0	0	7	0	0	0	0	0	0	0.2	0	0	0	0	0	0	0	0	0	3	0	1	6	3	1	0	0	0	0	0	3
Gait score (%)	0	0	0	0	0.6	0	0	0	0	0	0	0	0	0	0	0	0	0	0.3	0	2	0	0	0	0	0	1	1	0	0	0	0
Risk of injuries (n)	0	0	0	0	0	0	13	0	0	0	5	0	0	0	0	4	0	0	0	0	0	0	0	0	0	0	0	0	0	0	0	20
Coughing (n)	0	0	0	0	0	0	0	0	0	0	0	0	0	0	0	0	0	0	0	0	0	0	0	0	0	0	0	0	0	0	0	0
Sneezing (n)	0	0	0	0	0	0	0	0	0	0	3	0	0	0	0	0	0	0	0	0	2	3	0	2	1	7	1	1	7	0	0	3
Nasal discharge (%)	0	0	0	0	0	0	0	0	0	0	0	0	0	0	0	0	0	0	0	0	0	0	0	0	0	5	0	0	0	0	0	5
Ocular discharge (%)	0	0	0	0	0	0	0	0	0	0	0	0	0	0	0	0	0	0	0	0	0	0	0	0	1	1	0	0	0	0	0	3
Skin condition (%)	0	0	0	0.5	0	0	0	0	0	0	3	0	7	0	2.7	0	0	0	0	0	74	34	13	28	47	1	1	50	52	1	64	90
Neck torsions (%)	0	0	0	0	0	0	0	0	0	0.5	0	0	0	0	0	0	0	0	0	0	1	1	2	0	0	2	0	2	2	0	0	0
Cleanliness (0–1–2)	0	0	0	0	0	0	0	0	0	0	0	0	0	0	0	0	0	0	0	0	1	1	0	1	1	0	1	1	1	1	1	2
Flies presence (0–1–2)	0	0	0	0	2	0	2	0	2	2	2	2	2	0	0	2	0	0	0	0	0	0	0	0	0	0	0	0	0	0	0	0
Killing methods (0–2)	2	2	2	2	2	2	2	2	2	2	0	2	2	2	2	2	2	2	2	2	2	2	2	2	2	2	2	2	2	2	2	2
Negative social (%)	0	0	0	0	0	0	0	0	0	0	0	0	0	0	0	0	0	0	0	0	0	0	0	0	0	0	0	0	0	0	0	0
Isolated animals (%)	0	0	0	0	0	0	0	0	0	0	0	0	0	0	0	0	0	0	0	0	0	0	0	0	0	0	0	0	0	0	0	0
Abnormal b. (%)	0	0	0	0	0	0	0	0	0	0	0	0	0	0	0	0	0	0	0	0	0	0	0	0	0	0	0	0	0	0	0	13
Training (0–1–2)	2	2	2	2	2	0	2	2	2	2	1	2	2	2	0	2	2	1	2	0	2	2	2	2	2	2	2	2	2	2	2	2

## References

[1] EU D.G. Health and Food Safety (2016). Special Eurobarometer 442: Attitudes of Europeans towards Animal Welfare.

[2] Food and Agriculture Organization of the United Nations (2020). FAOSTAT Food and Agriculture Data. http://www.fao.org/faostat/en/#data.

[3] Blokhuis H., Veissier I., Miele M., Jones D.B. (2010). The welfare quality project and beyond: Safeguarding farm animal well-being. Acta Agric. Scand. Sect. A Anim. Sci..

[4] De Jong I.C., Reuvekamp B.F.J., Rommers J.M. (2011). A Welfare Assessment Protocol for Commercially Housed Rabbits, Report 532.

[5] Hansen S.W., Moller S.H. (2001). The application of a temperament test on-farm selection of mink. Acta Agric. Scand. Sect. A Anim. Sci..

[6] Welfare Quality^®^ (2009). Welfare Assessment Protocol for Pigs.

[7] Welfare Quality^®^ (2009). Welfare Assessment Protocol for Poultry.

[8] Popescu S., Diujan E.A., Borda C., Mahdy C.E. (2013). Welfare assessment of farmed rabbits housed in indoor and outdoor cages. Sci. Paper Anim. Sci. Biotechnol..

[9] Morton D., Verga M., Blasco A., Cavani C., Gavazza A., Maertens L., Szendro Z. (2005). The Impact of the current housing and husbandry systems on the health and welfare of farmed domestic rabbits. EFSA J..

[10] Welfare Quality^®^ (2009). Welfare Assessment Protocol for Cattle.

[11] Turner P., Buijs S., Rommers J.M., Tessier M. (2018). Code of Practice for the Care and Handling of Rabbits: Review of Scientific Research on Priority Issues.

[12] Verga M., Luzi F., Szendro Z., Maertens L., Coudert P. (2006). Behavior of growing rabbits. Recent Advances in Rabbit Sciences.

[13] Kells N.J., Beausoleil N.J., Cogger N., Johnson C.B., O’Connor C., Webster J., Laven R. (2020). Indicators of dehydration in healthy 4–5 day-old calves deprived of feed and water for 24 hours. J. Dairy Sci..

[14] Lebas F., Coudert P., de Rochambeau H., Thébault R.G. (1997). The Rabbit—Husbandry, Health and Production.

[15] Amici A., Canganella F., Bevilaqua L. (1998). Effects of high ambient temperature in rabbits: Metabolic changes, caecal fermentation and bacterial flora. World Rabbit Sci..

[16] Franci O., Amici A., Margant R., Merendino N., Piccolella E. (1996). Influence of thermal and dietary stress on immune response of rabbits. J. Anim. Sci..

[17] Dalmau A., Catanese B., Rafel O., Rodriguez P., Fuentes C., Llonch P., Mainau E., Velarde A., Ramon J., Taberner E. (2015). Effect of high temperatures on breeding rabbit behaviour. Anim. Prod. Sci..

[18] Marai I.F.M., Habeeb A.A.M., Gad A.E. (2002). Rabbits’ productive, reproductive and physiological traits as affected by heat stress: A review. Livest. Prod. Sci..

[19] Trocino A., Xiccato G. (2006). Animal welfare in reared rabbits: A review with emphasis on housing systems. World Rabbit Sci..

[20] Szendro Z. (2009). The Relationship between Housing Systems and Animal Welfare. http://www.asic-wrsa.it/documenti/giornate2009/03_Szendro.pdf.

[21] Verga M., Luzi F., Petracci M., Cavani C. (2009). Welfare aspects in rabbit rearing and transport. Ital. J. Anim. Sci..

[22] EFSA AHAW Panel (EFSA Panel on Animal Health and Welfare) (2020). Scientific Opinion on the health and welfare of rabbits farmed in different production systems. EFSA J..

[23] White S.D., Bourdeau P.J., Meredith A. (2002). Dermatologic problems of rabbits. Semin. Avian Exot. Pet Med..

[24] Rauterberg S.L., Bill J., Kimm S., Kemper N., Fels M. (2019). Effect of a new housing system on skin lesions, performance and soiling of fattening rabbits: A German case study. Animals.

[25] Buseth M.E., Sauders R. (2015). Rabbit Behaviour, Health and Care.

[26] Harkness J.E., Turner P.V., VandeWoude S., Wheler C.L. (2010). Biology and husbandry. Harkness and Wagner’s Biology and Medicine of Rabbits and Rodents.

[27] Dalmau A., Moles X., Pallisera J. (2020). Animal welfare assessment protocol for does, bucks and kit rabbits reared for production. Front. Vet. Sci..

[28] Saxmose Nielsen S., Alvarez J., Bicout D.J., Calistri P., Depner K., Michel V., EFSA Panel on Animal Health and Welfare (AHAW) (2020). Scientific opinion concerning the killing of rabbits for purposes other than slaughter. EFSA J..

[29] Lidfors L., Edstrom T., Lindberg L. (2007). The welfare of laboratory rabbits. The Welfare of Laboratory Animals.

[30] Rommers J., Meijerhof R. (1998). Effect of group size on performance, bone strength and skin lesions of meat rabbits housed under commercial conditions. World Rabbit Sci..

[31] Dalmau A., Abdel-Khalek A.M., Ramon J., Piles M., Sanchez J.P., Velarde A., Rafel O. (2015). Comparison of behaviour, performance and mortality in restricted and *ad libitum* fed growing rabbits. Animal.

[32] Verga M., Carenzi C. (1981). Il Comportamento Degli Animali Domestici.

[33] Lawrence A.B., Rushen J. (1993). Stereotypic Animal Behaviour: Fundamentals and Applications to Welfare.

[34] Chu L., Garner J.P., Mench J.A. (2004). A behavioral comparison of New Zealand White rabbits (Oryctolagus cuniculus) housed individually or in pairs in coventional laboratory cages. Appl. Anim. Behav. Sci..

[35] Gunn D., Morton D.B. (1995). Inventory of the behavior of New Zealand White rabbits in laboratory cages. Appl. Anim. Behav. Sci..

[36] Bozicovich T.F.M., Moura A., Fernandes S., Oliveira A.A., Siqueira E.R.S. (2016). Effect of environment enrichment and composition of the social group on the behaviour, welfare, and relative brain weight of growing rabbits. Appl. Anim. Behav. Sci..

[37] Buijs S., Keeling L.J., Tuyttens F.A.M. (2011). Behaviour and use of space in fattening rabbits as influenced by cage size and enrichment. Appl. Anim. Behav. Sci..

[38] Rushen J., Passillé A.M., Grandin T. (2010). The importance of good stockmanship and its benefits for the animals. Improving Animal Welfare: A Practical Approach.

